# The effect of multiple natural enemies on a shared herbivore prey

**DOI:** 10.1002/ece3.5451

**Published:** 2019-07-17

**Authors:** Maartje J. Klapwijk

**Affiliations:** ^1^ Department of Ecology Swedish University of Agricultural Sciences Uppsala Sweden

**Keywords:** co‐existence, complementarity, metapopulation, population dynamics, predator‐prey, redundancy

## Abstract

1. Natural enemy diversity is thought to be important for effective suppression of herbivores in production systems. Studies investigating the importance of the diversity and composition of the natural enemy complex often use within‐year empirical studies or experimental exclusion setups.
2. However, within‐year population suppression might not translate in long‐term population regulation. Therefore, I used a combination of long‐term data collection and an exclusion experiment to investigate mechanisms behind year‐to‐year population changes and potential effects of disturbance of the natural enemy complex.
3. Using the holly leaf miner study system in Wytham Woods, I find that the dominant predator in the system does not necessarily contribute the most to the reduction in year‐to‐year changes in mine density or within‐patch fluctuations. Using the exclusion experiment, it becomes clear that parasitism later in the prey life cycle can to a certain level compensate for disruption of mortality in the earlier life stage of the prey.
4. Thus, for host suppression in perennial systems the mortality pressure over the whole life cycle is important and disturbance during one part of the life cycle might not necessarily be buffered by mortality in other parts of the life cycle, especially if the natural enemy complex consists of multiple predator guilds.

## 1 INTRODUCTION

Evidence that increased diversity of natural enemies will lead to reduced fluctuations in herbivore densities is mounting (Snyder, Snyder, Finke, & Straub, 2006; Straub & Snyder, 2008) but not unequivocal (reviewed by Letourneau, Jedlicka, Bothwell, & Moreno, 2009; Macfadyen et al., 2009). Higher natural enemy diversity is thought to also lead to functional redundancy within the natural enemy complex (Loreau, 2004), which will maintain stable suppression over time. Thus, species are thought to respond differently to disturbances and if one species is rare 1 year, another species will be more abundant (Yachi & Loreau, 1999). However, a review of experimental studies shows that several directions of the outcome of interactions between predators can be expected (Schmitz, 2007). More recent studies doubt the importance of intraguild predation or facilitation between predators in determining the outcome in multiple predators—shared prey systems (Northfield, Crowder, Takizawa, & Snyder, 2014; Roubinet et al., 2015). Field experiments often focus on within‐year population growth rates and discuss suppression at that time scale, for example, within agricultural systems (Dainese, Schneider, Krauss, & Steffan‐Dewenter, 2017). Utilization of hosts in different life stages can be seen as resource partitioning (Briggs, 1993; Northfield, Snyder, Ives, & Snyder, 2010), and principles are applied in biological control (Schoellerl & Redak, 2018). In this study, I investigate the effects of a diverse enemy complex on a stage‐structured host and potential complementary between enemies attacking the host in different life stages.

The aim of the presented work was to use a well‐known study system, the holly leaf miner (*Phytomyza ilicis*), to investigate the role of predators attacking a shared prey at different times in the host life cycle. To estimate the role over time, I assess the effect of predation mortality on the year‐to‐year rate of change of the host population in a patch. For this analysis, I use a 9‐year data collection on mine density, survival and predation mortality inflicted by predators from different functional groups. In addition, an exclusion experiment was applied to investigate whether increased survival of the host in one life stage could be compensated by higher mortality rates inflected in later life stages.

Year‐to‐year changes in holly leaf miner densities are dependent on oviposition rates, egg survival, larval and pupal survival, and adult dispersal success. Previous studies on the relationship between patch properties, population fluctuations, and two types of parasitism rates, larval and pupal parasitism, showed that larval parasitism rates respond positively to mine density in a patch, whereas this response is not observed for the pupal parasitism rates (Klapwijk & Lewis, 2012). In addition, identification of the species in the complex of parasitoids attacking the holly leaf miner found that one species, attacking during the larval stage, is the dominant parasitoid in the system. The parasitoid appears to have competitive advantage as it attacks the holly leaf miner larvae in an early stage in the life cycle. The pupal parasitoids are left with hosts that have successfully pupated, that is, those that were not attacked by the larval parasitoid (Klapwijk & Lewis, 2011).

Using the time series, yearly changes in mine densities can be calculated and related to predation inflicted in the different life stages of the host. The yearly changes in mine densities represent a coarse measure for population growth, and relating these to predation will give insight in the effect of predation in different life stages on within‐year population change. The variability in mine densities in the individual patches (trees) can be calculated and contributed to the mortality inflicted in different life stages. Calculating within‐patch fluctuations and relating these to predation will give insight in the contribution to population stability/fluctuations within a patch over time.

In order to stabilize the host population, predation during different life stages should ideally be complementary. One could expect that the natural enemy contributing the strongest to the year‐to‐year reduction in the rate of change could also have the largest effect on the reduction on overall fluctuations of the host population within a patch. But if the inflicted predation rates during different life stage are complementary, every natural enemy will contribute to reduced fluctuation, that is, influence within‐patch host stability positively (reduce fluctuations). This translates in three questions: (a) How does mortality inflicted by different predators affect yearly changes in mine density? (b) How do the predators contribute to within‐patch fluctuations in mine densities and thus to potential population stability?, and (c) Is a reduction in mortality inflicted during an earlier life stage compensated by mortality in the later life stage of the host?

## 2 METHODS

### 2.1 Study system

**Figure 1:**
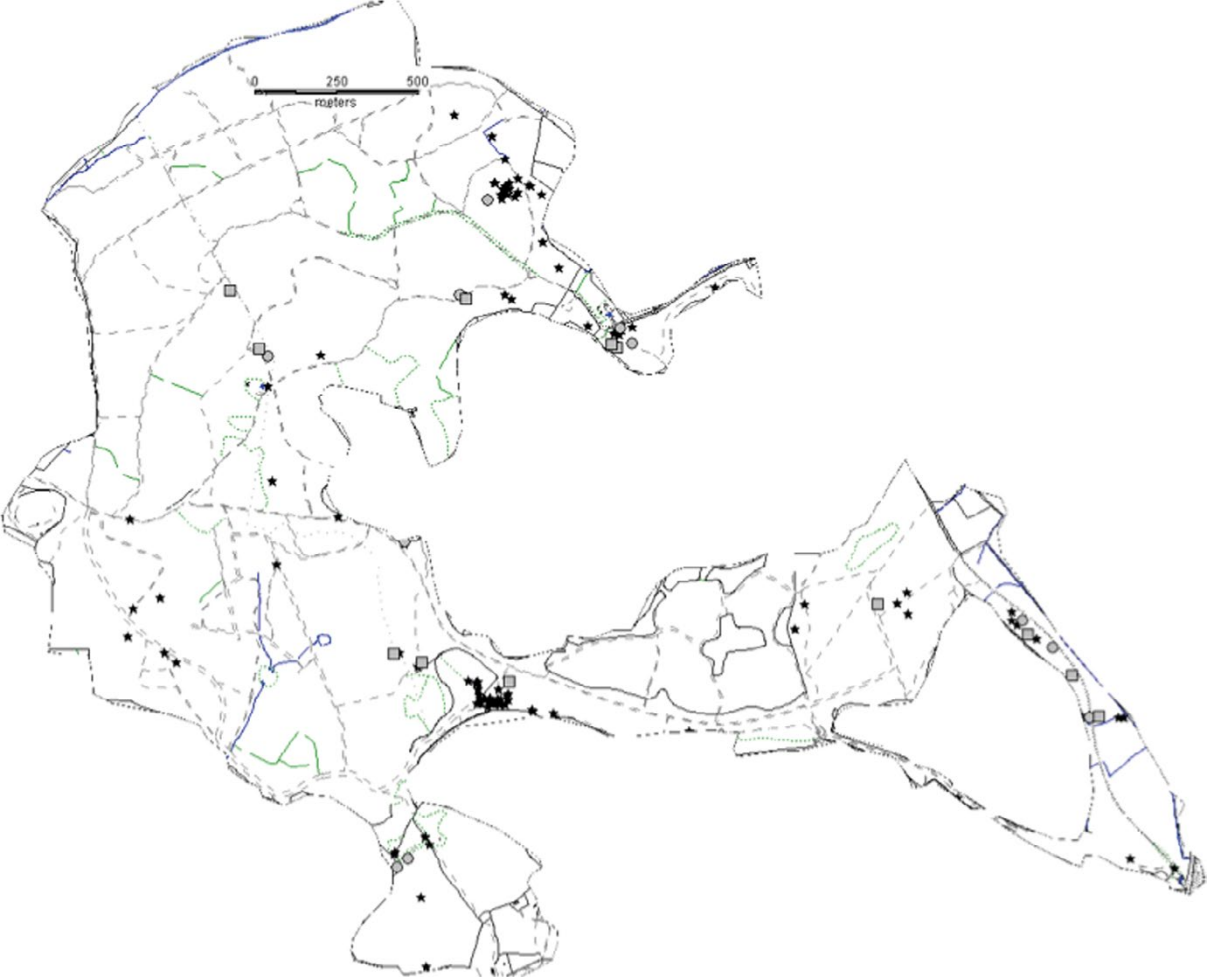
Wytham Woods, Oxford, UK. The gray rectangles represent the netted (covered) patches, and the gray circles are the symbols for the control patches (exposed trees). The black stars are the other marked holly patches in Wytham Woods

The holly leaf miner, *Phytomyza ilicis* (Curtis, 1948) (Diptera; Agromyzidae), and parasitoid complex is a classical system for ecological research (Brewer & Gaston, 2002, 2003; Cameron, 1939; Eber, 2001, 2004; Gaston, Genney, Thurlow, & Hartley, 2004; Klok, Chown, & Gaston, 2003; Lewis & Taylor, 1967). The species is a specialist herbivore of the holly tree*, Ilex aquifolium* L., and common throughout the UK and most of Europe. This leaf miner is univoltine, laying its eggs in the newly flushed leaves. The larvae live near the mid‐rib until late autumn when they start creating distinctive blotch mines. Holly is evergreen, and the larvae feed over winter and pupate in early spring. The study site is Wytham Woods near Oxford, UK (SP468085), an area of 415 ha comprising a mixture of deciduous woodland and plantation with open grassland areas. The holly trees at this locality have a scattered spatial distribution, representing a patchy resource for the host (Klapwijk & Lewis, 2012; Figure 1). In total, the identified patch network consisted of 163 individual trees over largely varying size and isolation. Patches within the Wytham Woods network are isolated by 1 km from other holly trees.

Egg survival is strongly density‐dependent; on average, only 40% of all eggs and young larvae will develop into a mine and during these early stages, mortality is largely caused by interspecific competition rather than predation (Brewer & Gaston, 2003; Eber, 2004; Valladares & Lawton, 1991). The eggs and early larval stages will be attacked by two parasitic wasps, *Chrysocharis gemma* and *Opius ilicis,* the first species is a generalist, and the second species is thought to be a specialist parasitoid (Cameron, 1939, 1941). During the winter months, after the leaf miner larvae have started to form blotch mines on the upper side of the leaf, the larvae are exposed to predation by birds. After pupation, there are three other common parasitoids that lay eggs in or on the pupae inside the mines, *Chrysocharis pubicornis*, *Cyrtogaster vulgaris,* and *Spegigaster flavicornis* (Klapwijk & Lewis, 2011; Lewis & Taylor, 1967), and these species are considered generalist parasitoids. One parasitoid species, *C. gemma*, dominates the parasitism rates (Brewer & Gaston, 2003; Valladares & Lawton, 1991), and this parasitoid species attacks early larval stages of the leaf miner. In the holly patch network in Wytham Woods, the species is present in the majority of patches and shows negative interactions with the presence and parasitism rates of the other parasitoid species (Klapwijk & Lewis, 2011). For the generalist parasitoid species, the holly leaf miner is likely to be the most important host in woodland habitats where other potential dipteran leaf miner hosts are scarce during early spring.

### 2.2 Collection and rearing of samples

The fate of each leaf miner individual can be determined by examining vacated mines in early summer. Hence, holly leaf miner population sizes, bird predation, and attack rates partitioned among associated parasitoids can be quantified without destructive sampling (Klapwijk & Lewis, 2012). These data were collected from 2002 to 2010. Mortality was classed in four categories, parasitoid emerged from larvae, parasitoid emerged from pupae, bird predation, and miscellaneous death, and in addition, host survival was recorded. For each tree, approx. 50 mined leaves were collected and used to determine the source of mortality through dissection of the mines. To establish mine densities within a patch, 10 branches were haphazardly selected, the total number of leaves was counted, and the number of mined and un‐mined leaves was recorded. In 2010, collected mines were reared in the laboratory (Klapwijk & Lewis, 2011), and after emergence, the individuals were registered and frozen. Identification was done at a later stage using identification methods and voucher samples from previous rearing (Klapwijk & Lewis, 2011).

### 2.3 Exclusion experiment

In order to investigate the contributions of the natural enemies to mortality inflicted during different life stages of the shared host, an exclusion experiment was used. For the experiment, trees of roughly the same size and “isolation” were selected. The experiment was set up after oviposition but before leaf miner larval feeding starts to be visible in the typical blotch mines on the leaf surface. Two trees were too large to be covered by netting; hence, their shape was adjusted by cutting off top branches, check afterward did not show that this affected the outcome of the experiment. The timing of experimental setup meant that all enemies during the overwintering period were excluded from the patches, larval parasitism, bird predation, and potentially sources of miscellaneous deaths.

Fine mesh netting (1.35 mm standard mesh insect netting) was used to exclude the larval parasitoids. The mesh netting was kept in place by tent pegs and sand at ground level. Thirteen trees to cover were selected, and 13 trees were selected to function as control patches; all selected patches were within a similar size and isolation range. The replicates were spread evenly through the woods and ranged from isolated trees to trees in aggregation of holly trees (Figure 1). As the holly leaf miner is univoltine, the exclusion treatment was setup in September 2009 and removed in the beginning of April 2010.

### 2.4 Data handling

I used the data collected from 2002 to 2010 to relate predation to yearly density changes of the leaf miner and the magnitude of within‐patch fluctuations. For the analyses of the exclusion experiment, the data collected in 2010 were used, both data on the fate of each mine and parasitoid identifications. The main type of analyses used is general (mixed) linear models (GL(M)M). All analyses were carried out using R 3.3.0 (R Core Team, 2018) for Mac.

The mine densities are calculated by dividing the number of leaves with mines by the total number of leaves counted. The rate of change between years for mine densities is calculated by subtracting the mine densities of the previous year (year *t*) from the mine densities of the current year (year *t* + 1; thus, rate of change = mine density*_t_*_+1_ − mine density*_t_*). Host survival is calculated as proportion of the total mined leaves collected. Bird predation is calculated as the number of leaves with mines that were cut open by a bird beak divided by the total number of mined leaves collected. Miscellaneous death is the proportion of leaves with undeveloped mines of the total collected mined leaves. Larval and pupal parasitism rates are calculated by dividing the number of determined mines by the total successfully hatched individuals (leaf miner fly and parasitoid). In order to minimize the influence of small and large proportion, we excluded patches smaller than 25 branches and samples where the number of leaves with mines counted was 10 or smaller. A summary of the calculations for each proportion variable is given in Table 1, together with the overall average and standard error for each variable.

**Table 1:** Summary of the variables calculated from the collected data on mine density, survival, and mortality. The values represent the average mean values with the standard error over the whole data set (all years and patches; 163 patches and 9 years)

Variable	Data use	Mean ± *SE*
Mine density	Counted mined leaves/Total number of counted leaves on 10 branches	0.11 ± 0.03
Rate of change	Mine density*_t_*_+1_ – mine density_t_	
Host Survival	Hatched leaf miners/total collected mined leaves	0.21 ± 0.03
Bird predation	Leaves with beak marks/total collected mined leaves	0.16 ± 0.04
Miscellaneous death	Undeveloped mines/ total collected mined leaves	0.25 ± 0.03
Larval parasitism rates	Number of parasitoids from larvae/total collected mined leaves (Number of parasitoids from larvae/number of hatched individuals)	0.24 ± 0.03 (0.42 ± 0.04)
Pupal parasitism rates	Number of parasitoids from pupae/total collected mined leaves (Number of parasitoids from larvae/number of hatched individuals)	0.12 ± 0.04 (0.22 ± 0.04)

### 2.5 Statistical analyses

#### 2.5.1 Patterns over time; predation; and year‐to‐year change

The relationships between year‐to‐year changes in mine densities (rate of change), host survival, and mortality are established using three separate generalized linear mixed regression with Gaussian quasi‐likelihood distribution. The first model contained the response variable rate of change and explanatory variables mine density in the previous year (year *t*), patch size (log_e_), and connectivity (Klapwijk & Lewis, 2011, 2012; Moilanen & Nieminen, 2002). The second model has the same response variable, and the explanatory variables were host survival at year *t*, patch size, and connectivity. The third model contained rate of change as response variable and miscellaneous death rates, bird predation, larval and pupal parasitism rates at year *t*, and their interactions. Backwards model simplification was used to obtain the minimal adequate model (Crawley, 2012). For these analyses, only patches that are measured all nine years are included (*n* = 29), patch identity is included as random variable to account for the yearly repeated measures and mine densities year *t*, patch size, and connectivity can be interpreted as continuous blocking factors.

#### 2.5.2 Patterns over time; within‐patch fluctuations

First, to obtain an accurate estimation of average mine density and within‐patch variability, I used a generalized linear model with quasi‐binomial error structure. In the model, mine density was the response variable and the explanatory variable was patch identity. I extracted the estimated mean and standard error of the estimate from the model output. The same procedure was used to estimate the mean for each explanatory variable, miscellaneous death, bird predations, larval parasitism rates, and pupal parasitism rates. Patches with an average mine density below 0.05 were excluded from the analysis because of the disproportional effect of low and irregular occupation on the standard error of the estimate (*n* = 97).

Subsequently, I formulated the model to estimate the relationship between standard error of the estimate of mean mine density (response) and mean host survival (explanatory variable) adding patch characteristics, that is, patch size (log_e_) and connectivity. In the next model, I assessed the relationship between the standard error of the mean for mine density and the mean mortality rates by the natural enemies. Hence, I applied a GLM with variation in mine density as response variable and as explanatory variables the mean of bird predation, larval parasitism, pupal parasitism, and miscellaneous deaths and their interactions. Because the model variables were correlated, extra care was taken using the variables in one model. Each variable was tested individually, and these values were compared to the model variables. Again, I used backward model simplification was used to obtain the minimal adequate model.

### 2.6 Exclusion experiment

A binomial GLM is used to assess the overall effect of exclusion on the contribution to bird predation, miscellaneous death, larval parasitism, and pupal parasitism. I used a binomial GLM to test the differences between the two manipulation levels in host survival. In a second model, the response variable in the GLM was total proportion mortality and the explanatory variables were manipulation (levels: exposed and covered), source of mortality (4 levels: miscellaneous deaths, bird predation, larval parasitism rates, and pupal parasitism rates), and the interaction between the two main effects. I also used overall parasitism rates in 2010 to compare the parasitism rates between covered and exposed patches (*n* = 24).

Individual responses to the exclusion treatment were assessed for *Chrysocharis pubicornis, Chyrtogaster vulgaris, Opius ilicis, and Sphegigaster flavicornis*. To compare the parasitism rates of the separate species, I used a generalized linear model using penalized quasi‐likelihood to adjust for over‐dispersion. Patch identity was included as a random grouping factor. For each separate GLM(ER), the significance of the differences was calculated using a type II ANOVA (car—package) in which the chi‐square values are calculated using adjusted sums of squares (Fox & Weisberg, 2010), as some of the variation in the response variables was shared by several explanatory variables (i.e., the data are non‐orthogonal).

## 3 RESULTS

### 3.1 Year‐to‐year changes in mine density

**Figure 2:**
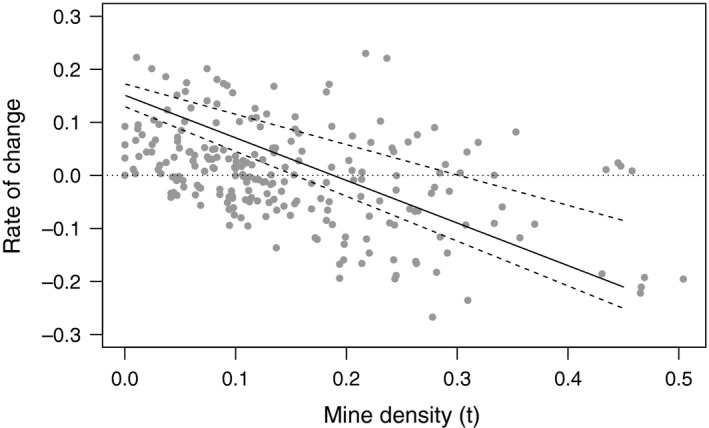
Relationship between the rate of change in mine density (year *t* + 1 − year *t*) and mine density (year *t*). The solid line represents the predicted values for the mortality (Table 2), and the dashed lines represent the standard error of the estimates

The yearly rate of change is low or negative if the mine densities were high in the previous year (−0.45 ± 0.05, *t*‐value = −8.28, *p* < 0.0001). High host survival in the previous year results in a positive rate of change, thus higher mine density in the following year (0.12 ± 0.03, *t*‐value = 3.51, *p* = 0.0006). Patch size (log_e_) and connectivity do not significantly affect the rate of change. Investigating the different natural enemies, pupal parasitism turns out to have the strongest negative effect the rate of change (−0.16 ± 0.04), and larval parasitism has a less strong effect (−0.08 ± 0.03; Table 2 and Figure 2). Bird predation and miscellaneous death do not affect the rate of change significantly. No significant interactions were identified.

**Table 2:** Minimum adequate model as result of the multiple regression using Gaussian quasi‐likelihood distribution

Fixed effects	*t‐value*	*df*	Estimate	*SE*
(Intercept)	7.04***	1	0.15	0.02
Mine density	−8.02***	1	−0.48	0.06
Patch size	−0.07	1		
Connectivity	0.08	1		
Misc. mortality	−0.90	1		
Larval parasitism	−2.82**	1	−0.08	0.03
Pupal parasitism	−4.09***	1	−0.16	0.04
Bird predation	−0.54	1		

Random effects	Obs/No. patches	Intercept	Residual
Patch ID	217/29	0.033	0.216

Note

Backward elimination was used to obtain the minimum adequate model. The response variable is the rate of change of mine density between years (mine density*_t_*_+1_ − mine density*_t_*). All explanatory variables are taken at time *t*. All two‐way interactions were included in the model, higher order interactions were excluded as their biological significance is hard to interpret. Mine density*_t_*, patch size (log_e_), and connectivity were included as continuous blocking factors. Patch size and connectivity were scaled to improve the model fit. Patch ID was included as random grouping factor (*n* = 218).

*** <0.0001.

** 0.001.

* 0.01.

### 3.2 Within‐patch variability of mine densities

The within‐patch fluctuations showed a strong negative relationship with the mine densities in the patch (−0.80 ± 0.09 *t* = −8.48, *p* < 0.0001); therefore, the subsequent analyses were weighted for mine density. Host survival had no relationship to the within‐patch variation in mine densities (0.03 ± 0.07, *t* = 0.39, *p* = 0.69). When investigating effect of mortality on within‐patch fluctuations, the four‐way and three‐way interactions were included but found to be not significant and therefore removed from the model. Bird predation and pupal parasitism rates affect the variability negatively and appear to exacerbate each other's effect on within‐patch variability in mine density (Table 3). Larval parasitism rates and miscellaneous death do not significantly affect the within‐patch fluctuations.

**Table 3:** Minimum adequate model obtained by backward elimination using multiple regression analysis of yearly variation in mine densities in relation to patch characteristics, host survival, and mortality sources

Variables	*t*‐value	*df*	Estimate	*SE*
(intercept)	6.249***	1	0.33	0.05
Patch size (log_e_)	−1.704^°^	1	−0.01	0.01
Connectivity	0.475	1		
Miscellaneous death	−0.968	1		
Bird predation	−3.027**	1	−0.80	0.27
Pupal parasitism	−2.127*	1	−0.45	0.21
Larval parasitism	−0.414	1		
Bird predation × Pupal parasitism	2.829**	1	4.07	1.44

Note

Model statistics: Adj *R* ^2^ = 0.11; *F*_7,89_ = 2.717; *p* = 0.01; *n* = 97.

The means and standard error of the estimated mean are calculated using a binomial GLM with tree identity as explanatory factor using a quasi‐binomial error distribution. The variation in mine density is expressed as the standard error of the estimated mean. Scaled patch size (log_e_) and connectivity are included as continuous blocking factors. The mine density for each patch was included as weighing factor. All trees smaller than 25 branches and trees with zero mean host survival were removed prior to modeling the mean and the standard error of the estimate for each patch. Patches with average mine density under 0.5% were removed as the error of the estimate had the tendency to be disproportionally large. Mine density was included as weight variable to account for the relationship between the mean and the variation in the mean. The values given indicate the *t*‐values, and the asterisk indicates significance level.

*** *<*0.0001.

** 0.001.

* 0.01.

^°^ 0.05.

### 3.3 Exclusion experiment

**Figure 3:**
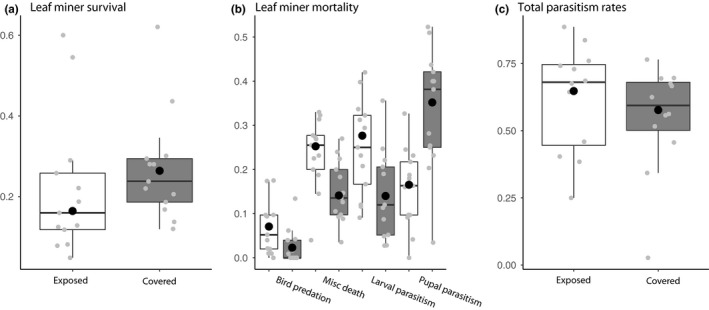
Boxplots for (a) Host Survival (b) Mortality Factors (c) Total parasitism rates. The gray dots represent the raw data. The large black dot in each boxplot represents the estimated mean for each box. The lower and upper hinges correspond to the first and third quartiles (the 25th and 75th percentiles). The upper whisker extends from the hinge to the largest value no further than 1.5 * interquartile range from the hinge. The lower whisker extends from the hinge to the smallest value at most 1.5 * IQR from the hinge (ggplot2 package—geom_boxplot; McGill, Tukey, Larsen, 1978)

Host survival was significantly higher in the manipulated patches (Table 4A; Figure 3a). Bird predation rates were lower in the trees that were covered with netting from September 2009 until April 2010, and the same was found for miscellaneous death rates and larval parasitism. The pupal parasitism rates were significantly higher in the covered patches, compared with the exposed patches (Table 4B and Figure 3b). The results show that total parasitism rates (larval and pupal parasitism rates summed) are not different between the covered and exposed patches (Table 4C and Figure 3c). All four parasitoid species increased their parasitism rates in patches that had been covered, but the increase was not significantly different between the different species (Table 5). The variance in the covered patches is higher compared with the variance of the exposed trees. But after model checking, it appeared that the variance is not significantly different for the treatment combinations.

**Table 4:** ANOVA table of the binomial generalized linear model with response (A) Host survival and (B) Proportion mortality. The estimates are given in logit; Figure 3 displays the variables in proportion. The contrast used for the estimates is the sum contrasts (see Crawley, 2012, p. 442 and 554) (C) Mixed model comparing overall parasitism rates in 2009 and 2010 considering patch identity as a random variable

	*χ* ^2^‐value	*df*	Level	Est ± *SE*
A. Host survival				
Manipulation	4.69*	1	Exposed	−1.62 ± 0.21
			Covered	−1.03 ± 0.18
B. Proportion mortality				
Mortality source	43.75***	3	Bird predation	−2.57 ± 0.25
			Misc. death	−1.09 ± 0.15
			Larval Par	−0.96 ± 0.14
			Pupal Par	−1.62 ± 0.18
Manipulation	6.31*	1	Covered	−1.18 ± 0.51
Mortality × Manipulation	48.09***	3	Misc. death × Covered	0.46 ± 0.56
			Larval × Covered	0.33 ± 0.56
			Pupal × Covered	2.19 ± 0.55
C. Overall parasitism				
Manipulation	1.22	1		

Note

The values given indicate the *χ* ^2^‐value*,* and the asterisk indicates significance level.

*** *<*0.0001.

** 0.001.

* 0.01.

**Table 5:** The results of the generalized linear model using penalized quasi‐likelihood with a binomial error structure to compare species response to the manipulation treatment with tree—identity included as a random effect

	*F‐*value	*df*	Levels	Estimate ±* SE*
Parasitism rates				
(Intercept)	73.83***	1	(Intercept)	−2.56 ± 0.31
Manipulation	4.94*	1	Covered	0.61 ± 0.28
Species	14.19**	3	*C. vulgaris*	0.33 ± 0.32
			*S. flavicornis*	−0.76 ± 0.40
			*O. ilicis*	−0.88 ± 0.44

Random	Obs/groups	(intercept)		Residual
Tree identity	96/24	4.54 × 10^–5^		2.34

Note

The estimates are based on the logit transformation for the proportion data. The values given indicate the *F*‐value*,* and the asterisk indicates significance level.

*** *<*0.0001.

** 0.001.

* 0.01.

## 4 DISCUSSION

Using 9 years of data collection, I showed that negative year‐to‐year changes in mine densities in a patch can be contributed more to pupal parasitism than to larval parasitism (Table 2). Within‐patch fluctuations of mine densities are mostly reduced by pupal parasitism rates in interaction with bird predation (Table 3). The exclusion experiment shows that pupal parasitoids have the ability to increase their inflicted parasitism rates when more vacant mines are available (Table 4C), but did not reduce host survival to the level of exposed patches (Table 4A).

In the holly system, pupal parasitoids attacking the leaf miner host in a later stage compared with the larval parasitoids should have superior host location ability within a patch, according to the theory of multiple parasitoids utilizing the same host (Briggs, 1993; Hassell, 2000). Suggesting that only if the parasitoid, attacking the host during a later life stage, has well‐developed searching behavior, the second parasitoid can persist in the system (Briggs, 1993). My results give an indication that this might be the case for the group of pupal parasitoids which provides a potential explanation why they had a stronger negative effect on the rate of change between two years compared with the larval parasitoids. At high mine densities, the pupal parasitoids locate those mines that have not been attacked by birds or larval parasitoids and manage to do this more efficiently at high densities compared with low densities.

The exclusion experiment resulted in higher host survival in the covered compared with exposed patches (Figure 3a). But the total parasitism rates remained at the same level (Figure 3c), not explaining the higher host survival. However, the exclusion experiment inadvertently excluded more than just the larval parasitoids, and it also reduced bird predation and miscellaneous deaths of leaf miners (Figure 3b). As a result, the availability of hosts during the pupal stage was higher than could have been those had not been excluded. Hence, the pupal parasitism rates increased but not efficiently enough to compensate for the reduction in mortality pressure earlier in the life stage. One explanation might be that even though within‐patch searching capacity is high, interference between individuals of the different species could result in reduced efficiency in host utilization (Visser, Jones, & Driessen, 1999). Thus, it appears that within groups of parasitoids the mortality of the host will be maintained at similar levels by the presence of multiple species, when mortality imposed by one of the species is disrupted (Naeem & Li, 1997; Yachi & Loreau, 1999) but only to a certain level. As insect herbivores are attacked by a multitude of different enemies during the different life stages understanding how relaxed mortality pressure during one life stage affects the survival, yearly change and long‐term fluctuations can be rather important to understand the long‐term effects of diversity on fluctuations in mortality by natural enemies.

Among patches, to inflict sufficient levels of mortality on their prey, predators should respond to prey densities (Abrams, 1982), especially in systems where the abundances of the prey have a large year‐to‐year variability. The pupal parasitoids as a group do not consistently positively respond to mine density in a patch (Klapwijk & Lewis, 2012). Each species responds individually (Klapwijk & Lewis, 2011), leading to variation in parasitism rates between years. Thus, among patches the inflicted parasitism rates are not related to mine density but a seemingly good host location ability enables the pupal parasitoids to reduce the mine densities in the next year. For the holly network in Wytham Woods, the between‐patch dynamics can be described as a patchy population structure for the leaf miners and the parasitoids (Klapwijk & Lewis, 2011; Thomas & Kunin, 1999). Especially within such patch network, for natural enemies to contribute to reduced population fluctuations they need to aggregate in patches with high host density (Hassell, May, Pacala, & Chesson, 1991; Vet, 2001). Even though the pupal parasitoids as a group do show a positive response to host density, they are instrumental in reducing year‐to‐year changes in mine density and the fluctuation within patches.

Hence, the results of this study confirm that spatial context (Tylianakis & Romo, 2010), ecology of the individual species, and the life stage in which they attack the host (Casula, Wilby, & Thomas, 2006) are important in the assessment of the individual contributions of natural enemies to host mortality and population suppression. Our results suggest complementarity of parasitism rates at different stages in the life cycle of a shared host. Local and regional level patterns might not be in the same direction (Wang & Loreau, 2016), and host location ability and host density responses of predators need to be considered at the relevant spatial scale.

The presented study focused on additivity or complementarity between parasitoids that consecutively attack the same host. In the system, bird predation also plays an important role in reducing the fluctuations in mine densities. To understand the effects of taxonomic distant predators, the experiment should have used more targeted exclusion regime to be able to quantify the separate and combined effects of all parasitism and bird predation. Especially as Griffin, Byrnes, and Cardinale (2013) suggest taxonomic distance between predators as explanation and found that average host suppression increased with the taxonomic distance between predators.

In conclusion, my results increase the understanding why diversity of parasitoids might not lead to increased host suppression confirming findings of other experimental and field studies (Rodriguez & Hawkins, 2000; Wilby, Villareal, Lan, Heong, & Thomas, 2005) and that complementary host use might lead to reduced host population variability even if not all species exhibit density‐dependent search patterns. Future studies investigating the effects of multiple predators on herbivore suppression should attempt to quantify the effects of different responses to host density (Vet, 2001) and spatial distribution of the herbivore host (Tylianakis & Romo, 2010) on local and regional population fluctuations.

## CONFLICT OF INTEREST

None declared.

## AUTHOR CONTRIBUTIONS

All work presented in this work is conceived, developed, carried out, analyzed, and written by MJK, who is the sole author of this work.

## Data Availability

The data have been archived using Dryad under https://doi.org/10.5061/dryad.67k2q1t
